# Dissecting the Chemical and Thermal Stabilities of Tetrads in G‐Quadruplexes to Derive a Structure‐Activity Relation for a Thrombin‐Binding DNA G‐Quadruplex Aptamer

**DOI:** 10.1002/cbic.202500743

**Published:** 2026-04-21

**Authors:** Julia Wirmer‐Bartoschek, Jan‐Peter Ferner, Alexander Heckel, Harald Schwalbe

**Affiliations:** ^1^ Institute for Organic Chemistry and Chemical Biology Center for Biomolecular Magnetic Resonance (BMRZ) Johann Wolfgang Goethe University Frankfurt am Main Germany; ^2^ Institute for Organic Chemistry and Chemical Biology Johann Wolfgang Goethe University Frankfurt am Main Germany

**Keywords:** G‐quadruplexes, hydrogen exchange, nuclear magnetic resonance (NMR) spectroscopy, nuclease resistance, thrombin binding aptamer

## Abstract

Guanosine‐ and deoxyguanosine‐rich nucleic acids can form G‐quadruplex structures (G4) that are stabilized by guanine tetrads (G4 tetrads). G4s find numerous applications in biotechnology. Here, we study a so called thrombin‐binding aptamer (TBA), developed by SELEX procedures, that adopts a G4 conformation and inhibits clotting of thrombin. We investigate the TBA G4 and its variants with either four adenosine desoxynucleotides or four abasic sites attached either to the 5′‐terminus (A4‐TBA and ab4‐TBA) or the 3′‐terminus (TBA‐ab4 and TBA‐A4). These variants have been shown to exhibit differential anticlotting activities previously. The variant TBA‐ab4, which was the most biological active in earlier investigations, has an exceptional stability against nuclease restriction, while all other variants show similar decay rates in mammalian serum. Biophysical characterization of the variants reveals that the structure of the aptamer remains unchanged, but that also their different thermal stabilities correlate with the anticlotting activity of TBA. Hydrogen exchange quantified by nuclear magnetic resonance spectroscopy (NMR) reveals individual G4 tetrad thermodynamics. Our data indicate that while enthalpy, entropy and free energy of base pair opening show surprisingly low variation, a hotspot for stabilization of the G4 is present at the 3′, 5′ terminal tetrad of TBA.

## Graphical abstract

Extended variants of the G‐quadruplex thrombin binding aptamer exhibit differential anticoagulation activities. We show that 3’ terminal extensions increase nuclease resistance, while variations in thermal stability directly impact activity. Hydrogen exchange nuclear magnetic resonance spectroscopy (NMR) report on thermodynamic properties of the base opening and reveals a key stabilization hotspot in the terminal tetrad.

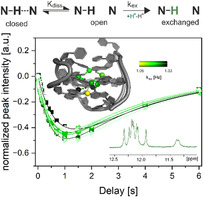

## 1 Introduction

Thrombin (factor IIa) is a serine protease that plays a key role in the coagulation process. It converts fibrinogen into fibrin, which forms the structural basis of blood clots, and activates platelets and other coagulation factors that amplify the clotting response [1]. The activity of thrombin is allosterically modulated by binding to two exosites: while exosite 1 provides an additional binding site for fibrinogen in addition to the active site, exosite 2 contains the heparin binding site. Both exosites are rich in basic amino acid residues that allow their binding to anionic substrates, ligands and inhibitors [2].

Inhibiting thrombin to prevent blood clotting remains a significant focus of research. Direct thrombin inhibitors can either bind to the active site or allosterically to the exosites. Known active site inhibitors, used in patients, are Bivalirudin, Lepirudin, and Argatroban [3]. Heparin is a widely used indirect thrombin inhibitor, binding to the exosite 2 and activating antithrombin amongst other cofactors to prevent clotting. However, it also interacts with a number of off‐target proteins, leading to side effects [4].

As an alternative approach to interfere with thrombin activity, the DNA aptamer thrombin‐binding aptamer (TBA) was developed by Bock et al. using SELEX [5]. It acts as anticoagulant targeting thrombin. TBA has been intensively studied [6]. The structure of the 15 nucleotide (5′‐GGTTGGTGTGGTTGG‐3′) minimal motif was solved by nuclear magnetic resonance (NMR) spectroscopy (148d.pdb). It consists of two G‐tetrads that are linked by two TT loops and one TGT loop, with the TT loops spanning the two narrow grooves of the G4 [7]. In addition, structures are available for the complex of TBA binding to thrombin (4DII.pdb, 4DIH.pdb, 3QLP.pdb) [8, 9]. Initial X‐ray structures provided ambiguous insight in the conformation and binding mode of TBA to thrombin [10, 11, 12], due to symmetry and hampered density in the loop regions of TBA. Meanwhile, based on higher resolution X‐ray structures, the bound conformation has been shown to be identical to the NMR solution structure of the free G4 [8, 9].

Binding of TBA to thrombin—with *K*_D_ between 0.25 [13] and 960 nM [14] determined by different groups using different buffer conditions and measurement techniques, reviewed in [15]—occurs at the fibrinogen binding site (exosite 1) involving the two TT‐loops of the aptamer. Binding inhibits the conversion of fibrinogen to fibrin. Initial preclinical studies showed TBA activity in monkeys, but later phase 1 clinical trials using TBA (ARC‐183) were stopped due to insufficient dose–response [16, 17]. Despite the termination of the clinical trials, optimization of the TBA aptamer to improve its role as a chemical probe continues to be of high interest. Current approaches include replacing nucleotides by either natural or modified nucleotides, changes of loop composition and size, changes in the sugar‐phoshodiester backbone, elongation or cyclization of the sequence, duplex‐quadruplex, homo‐ and heterodimer constructs [15]. Buff et al. [18] reported on the attachment of noncomplementary nucleotide extensions (A and T desoxynucleotides as well as abasic moieties). While 5′‐terminal extensions decreased the activity, 3′‐terminal extensions caused an increase in activity.

Here, we report the detailed characterization of the determinants of the stability changes of TBA variants at individual hydrogen bonds using NMR‐based hydrogen exchange data and thermal overall stability probed by circular dicroism (CD) spectroscopy. We further test the chemical stability of the TBA variants in mammalian serum. We investigate sequences with either four adenosine desoxynucleotides or four abasic sites attached either to the 5′‐terminus (**A**_**4**_‐**TBA** and **ab**_**4**_ **‐TBA**, respectively), or the 3′‐terminus (**TBA‐ab**_**4**_ and **TBA‐A**_**4**_) and compare them to the unmodified TBA. These variants had been reported to represent the most active (TBA‐ab_4_) and the least active (A_4_‐TBA) TBA variants [18].

## 2 Results and Discussion

### 2.1 All Variants Form Antiparallel G4 Structures

**FIGURE 1:**
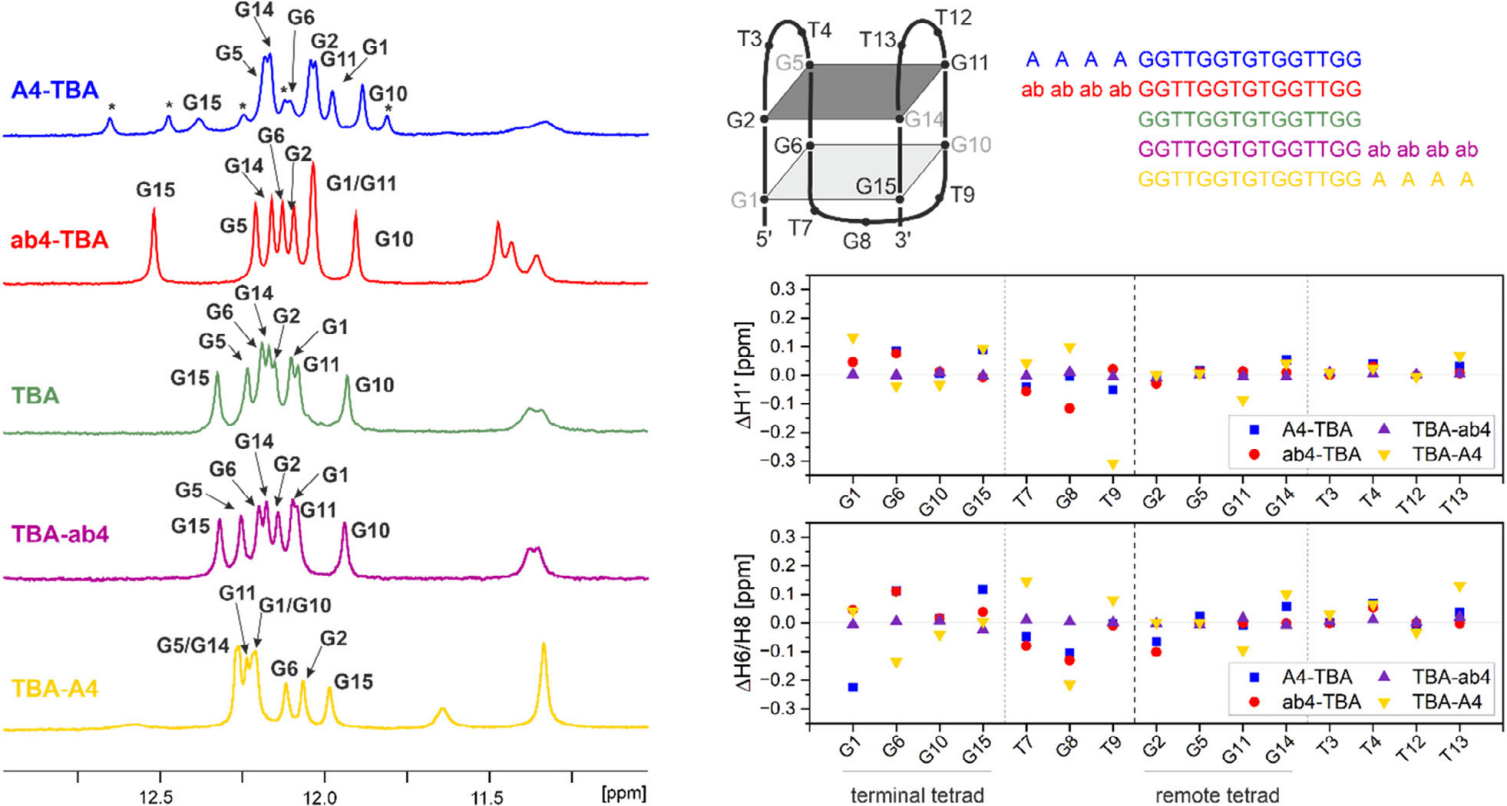
^1^H‐1D spectra (left) and schematic structure, sequences, and chemical shift deviations from TBA (right) of TBA variants at 278 K. Bases in the syn conformation are shown in gray, while antinucleotides are black. The remote tetrad is sketched in dark gray and the terminal tetrad in light gray. Signals stemming from a minor conformation are denoted with an asterisk *.

1D ^1^H NMR spectra at 278 K were recorded to evaluate whether the four TBA variants adopt a persistent G4 topology (Figure 1). For all investigated sequences, we detect imino signals between 11.7 and 13.0 ppm signifying the formation of G tetrads due to the unique chemical shifts of imino hydrogen atoms of nucleobases involved in persistent Hoogsteen interactions in G4's [19]. We further observe imino signals below 11.7 ppm accounting for hydrogen exchange‐protected imino protons of Ts and G8 in TBA [20]. The number of these latter NMR signals varies from construct to construct. The imino chemical shifts are remarkably different for the five samples.

**FIGURE 2:**
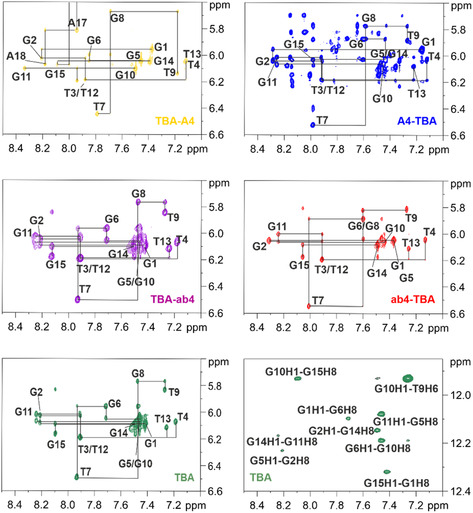
^1^H,^1^H NOESY spectra in D_2_O of the aromatic‐H1’ region of TBA and its variants at 278 K. In these spectra, each H6/H1’ and H8/H1’ crosspeak is labeled with its respective base. Interresidue crosspeaks are linked to their corresponding intraresidue crosspeaks to emphasize sequential connectivities. For TBA, an additional NOESY spectrum in H_2_O is provided, focused on the aromatic/imino region, where crosspeaks within the G‐tetrad are labeled to illustrate interactions within the tetrad structure.

We analyzed 2D NOESY experiments of TBA [7, 20] and its variants to assign the variants and to derive information about the χ angles of G desoxynucleotides involved in G4 formation. All variants share the same core structure: the χ angles of G1, G5, G10, and G14 adopt syn conformation, while all other desoxynucleotides populate anti conformation in all variants, as can be seen from the intensity or absence of H1’‐ aromatic crosspeaks (Figure 2). Characteristic imino–imino, imino‐aromatic (see Figure 2, lower right for TBA), and imino‐aliphatic cross peaks, reflecting on G‐quartet architecture as well as on loop architecture, are very similar in all variants. These data indicate that the overall conformation of the G4s is not changed in the variants compared to the original aptamer, and that all variants form antiparallel G4 structures in line with previous CD investigations (Figure S1) [18].

Analysis of imino chemical shift changes in the variants compared to the TBA aptamer allows more detailed analysis of conformational differences in the variants (Figure 1): Barely any variation from TBA is seen for the most active variant, TBA‐ab4. Variations in ab4‐TBA are small, with the exception of deoxynucleotide G15, which shifts almost 0.2 ppm downfield, indicating changes in G1‐G15 base pairing. Main differences are observed for TBA‐A4 and A4‐TBA. In A4‐TBA, additional signals accounting for a second, minor conformation, are observed and the T iminos are very broad, indicating conformational exchange. This exchange between the two folded conformations is slow at 278 K, so that we observe two stable states that do not interconvert on the NMR time scale. In line, we do not observe exchange cross peaks in NOESY spectra.

In TBA‐A4, significant chemical shift deviations of imino signals compared to TBA are observed in both G tetrads: G15 shifts more than 0.3 ppm upfield, while G10 is shifting almost 0.3 ppm downfield, moderate downfield shifts (0.1–0.16 ppm) are observed for G11 and G14. Further, a broad signal at 11.6 ppm appears, which could be assigned to G8. Similar small effects in all four variants are observed for sugar H1’ and aromatic H6/H8 protons, with larger variations in the terminal tetrad (G1‐G6‐G10‐G15) and the loop adjacent to the terminal tetrad than in the remote tetrad (G2‐G5‐G11‐G14) (Figure 1 right). Thus, chemical shift analysis shows changes within the terminal tetrad and the adjacent loop while the distances observed in NOESY experiments remain unaltered, pointing at more subtle effects in tetrad or overall stability.

### 2.2 Enhanced Stability against Nucleases in 3′ Modified Variants

**FIGURE 3:**
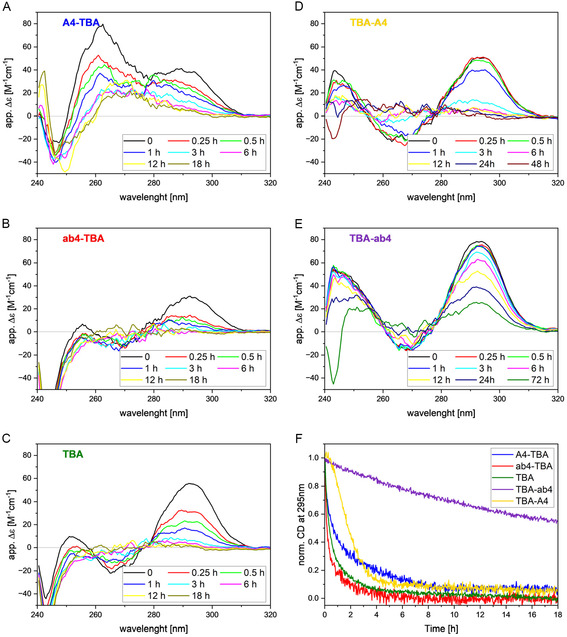
(A–E) CD spectra of TBA and its variants in 50% FBS at 310 K at different time points, monitoring the decay by nucleases. (F) Time‐dependent decay of the CD signal at 295 nm.

The stabilities of the TBA variants against degradation by nucleases were tested in fetal bovine serum (FBS) at 310 K (37°C) [21] to mimic the conditions of the coagulation assay. Figure 3 shows the results of CD measurements in a 1:1 mixture of FBS and Dulbecco's modified eagles medium (DMEM). At the start of the measurements (*t* = 0), all spectra show antiparallel G4 signatures (maximum at 295 nm, minimum around 267 nm), consistent with their behavior in NMR buffer ( Figure S1), but for A4‐TBA. Here, two maxima are detectable, one at 295 nm as observed in the antiparallel G4s and one at 260 nm, which could arise from a second presumably parallel conformation. We attribute this signature to a shift in the distribution of the two conformations visible also in the ^1^H NMR experiments above. The coexistence of at least two conformations in a more native environment could explain the reduced activity of the variant in hindering coagulation.

Normalized signal intensities at 295 nm monitoring the decay of the G4s are shown in Figure 3E. Rapid degradation is observed for TBA with less than 10% remaining signal intensity after 3 h. The decay of ab4‐TBA is slightly faster than that of the unmodified variant. The initial decay of A4‐TBA is the same for both visible bands (295 and 260 nm, see also Figure S2), reaching around 23% of the intensity after 3h, suggesting coexistence and exchange between both conformers under serum conditions. Signal decay of TBA‐A4 shows a lack phase of roughly half an hour (97% of the signal is still present after 30 min), before the signal decays fastly, going down to approximately 21% after 3 h. These data suggest that stability is enhanced by the presence of the four 3′ adenosines, which are presumably degraded sequentially by exonucleases without harming the overall structure before degradation of the G4 core starts. These findings are in line with earlier investigations, where modifications have also been introduced to increase the chemical stability of TBA by adding a 20 dA tail at the 3′ end, which enhances TBA's stability against degradation in human plasma and at the same time increases the clotting inhibitory activity [22].

The most active variant TBA‐ab4, is also the most stable variant in the nuclease assay. After 3 h, 85% of the signal corresponding to the folded G4 is present. Even after 72 h, a residual signal of an antiparallel G4 is present, with a signal intensity of around 25% at 295 nm could be detected. This implies that the 3′‐terminal abasic sites are less susceptible to degradation by nucleases, compared to 3′‐terminal desoxyadenosine additions. While the stability of the most active variant is striking, it has to be noted that the results of the nuclease assay cannot explain the differences in inhibiting coagulation [18] since the least active variant A4‐TBA is more resistant toward nucleases than the unmodified TBA. These data hint at an additional factor beyond chemical stability to explain differences in anticoagulation activity.

### 2.3 Thermal Stability Correlates with Activity

**FIGURE 4:**
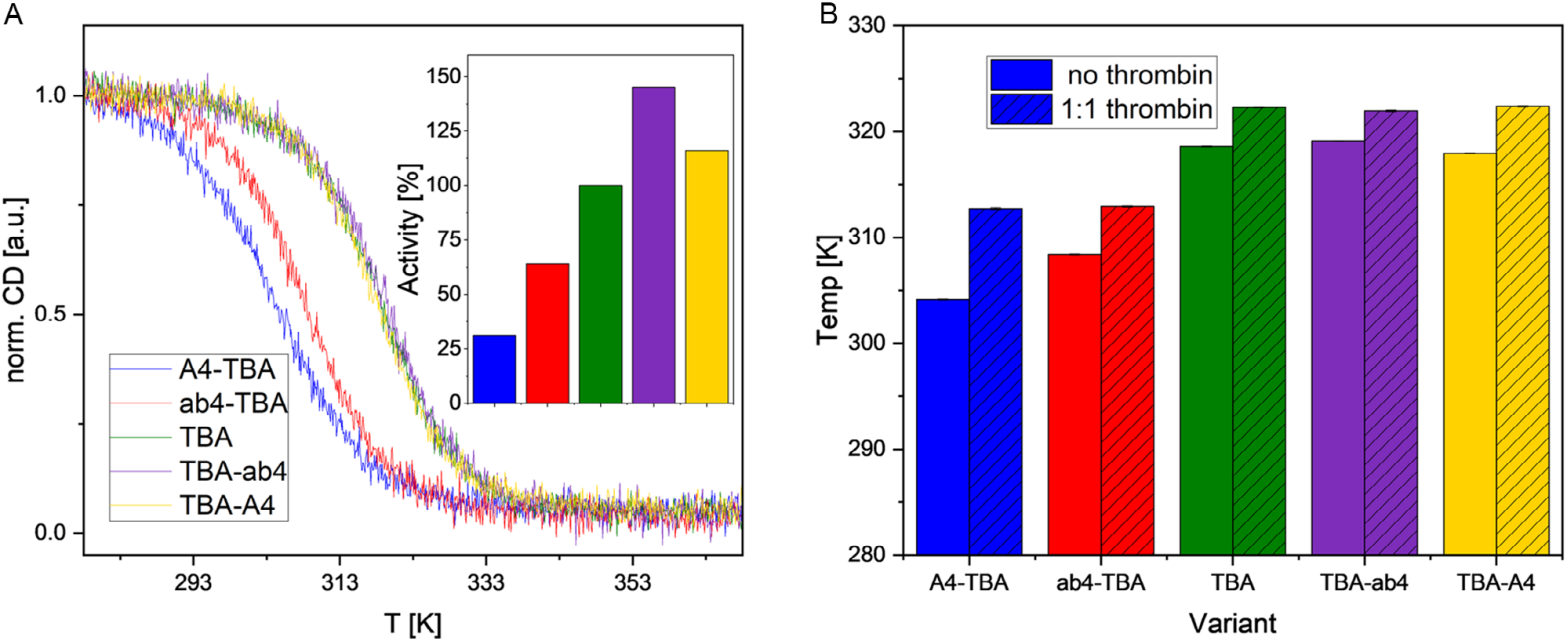
(A) Temperature‐dependent CD data at 260 nm in the absence of thrombin (normalized to 1), inlet shows activity of the respective variants, values are taken from Buff et al. [18]. (B) Summary of derived melting temperatures in the absence and presence of 1:1 thrombin.

Since the differences in the structure of the G4 are very small, and nuclease resistance lacks correlation with activity, we tested the thermal stability of the variants. Temperature‐dependent CD measurements of TBA reveal a melting point of 318.6 K for the unmodified TBA. This melting point is less than 10 K higher than the physiological temperature of clotting. This close proximity suggests a possible correlation between melting temperature and anticoagulation efficiency. Indeed, temperature‐dependent CD measurements show that TBA, along with TBA‐A4 and TBA‐ab4, have similar melting points around 318.6 K. In contrast, the melting points of ab4‐TBA and especially A4‐TBA are notably lower, indicating reduced thermal stability for these variants (Figure 4A).

The addition of thrombin increases the melting temperature across all TBA variants, reaching around 322.1 K (Δ*T*_m_ = 3.5 K) for TBA, TBA‐A4, and TBA‐ab4, and 312.8 K (Δ*T*_m_^ab4‐TBA^ = 4.5 K and Δ*T*_m_^A4‐TBA^ = 8.5 K) for the 5′ modified variants. The reduced anticoagulation activity [18] of ab4‐TBA and A4‐TBA appears to correlate with their lower thermal stability, resulting in a significantly smaller population of the folded state at physiological temperatures. These findings suggest that although the general structure of the variants remains unchanged, differences in global stability account for the observed variations in activity.

While it has been shown previously [18, 23] that addition of noncomplementary nucleotides to the 5′ end decreases the activity and unaltered or 3′ modified variants increase activity, fewer data are found that correlate the thermal stability with activity. De Rache et al. [23] monitored a decrease in melting temperature of noncomplementary 5′ extensions, which also decrease anticlotting activity, in line with our investigations. However, single nitroindole extensions have minimal impact on the melting temperature (*T*_m_^TBA^: 325.4 K, *T*_m_^N‐TBA^: 324.6 K, *T*_m_^TBA‐N^: 324.1 K) [24] and show no effect on clotting time. More complex modifications at both termini yield mixed outcomes. Tris‐mTBA, modified with a 3′dansyl residue and a 5′β‐cyclodextrin as well as a 5′ biotin, linker exhibits a significantly lower melting temperature than TBA (312 K vs. 322 K), however, a 10‐fold improvement in anticoagulation efficiency [25]. Riccardi et al. [26, 27] showed that cyclization of TBA in general increases thermal stability (e.g., *T*_m_^TBA^: 324 K *T*_m_^cycTBA^ variants: 325–342 K, depending on the linker) but does not consistently enhance anticoagulation activity. While cycTBA IV (*T*_m_ = 325 K, linker length 48 atoms) is more active, other variants exhibit significantly lower activity compared to unmodified TBA. Duplex‐TBA constructs such as NU172 and 31‐TBA in which complementary nucleotides are added to the 5′‐and the 3′ ends to keep the two ends of the G4 in proximity, have a significant higher anticlotting efficiency, while melting point of the G4 is slightly higher for 31‐TBA (315.9 K) and lower for NU172 (310.3 K) compared to TBA (312.3 K) [28, 29]

The results presented above indicate that nuclease resistance does not correlate with the melting temperatures of the variants. Although TBA and its 3‐modified variants exhibit similar melting temperatures, their nuclease stability differs markedly: TBA is degraded to below 45% within the first 30 min, TBA‐ab4 is highly stable and TBA‐A4 shows pronounced stability during the first 30 min, before rapid degradation starts. In contrast, the 5′‐modified variants, which display melting temperatures approximately 10 K lower than TBA, are degraded on a similar timescale as TBA. These observations suggest that nuclease degradation is primarily influenced by modifications at the 3‐terminus of the TBA G4 rather than by overall thermal stability.

### 2.4 Hydrogen Exchange Reveals Hotspot for Stabilization of the G4

**FIGURE 5:**
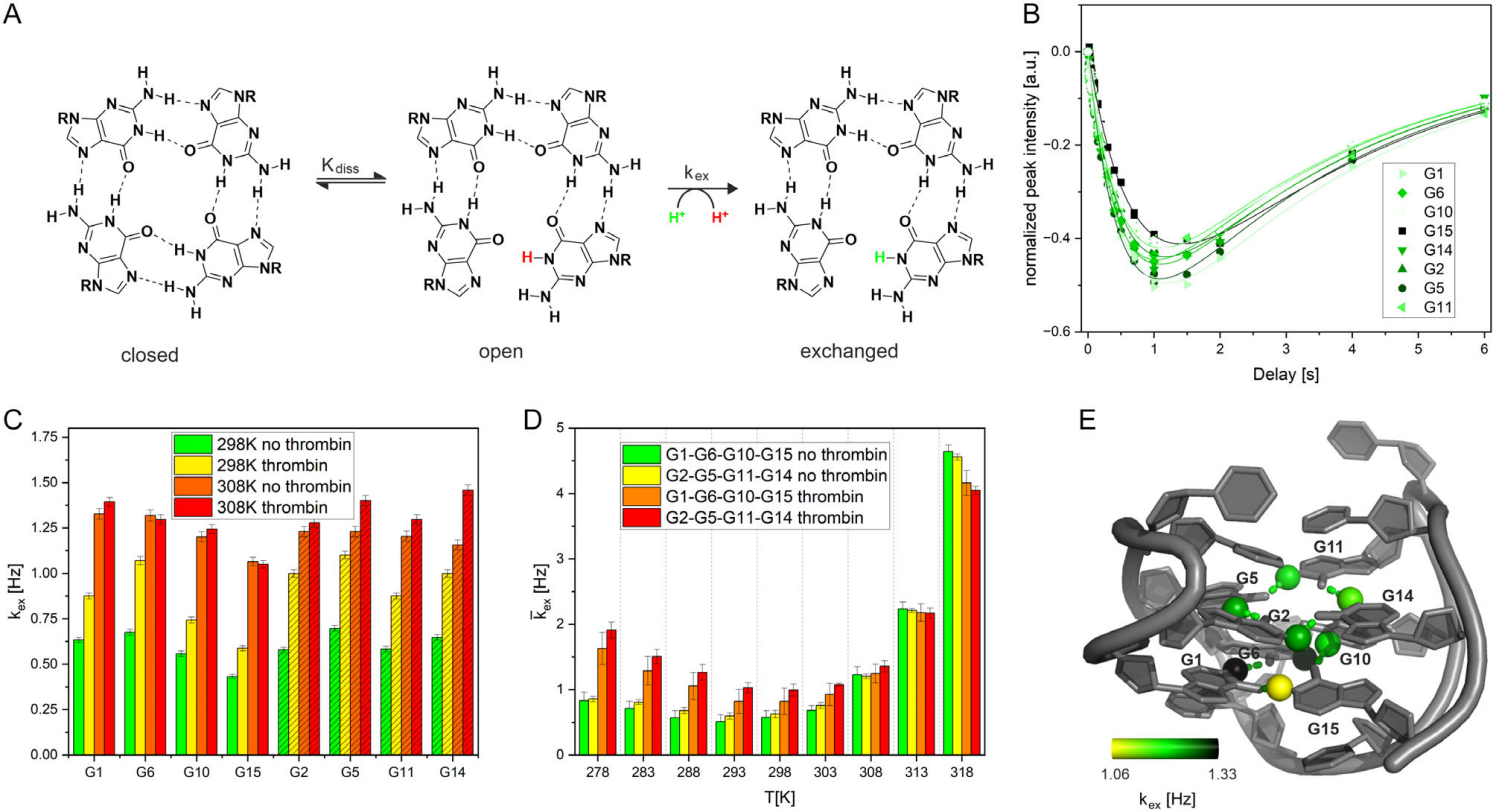
Hydrogen exchange rates of TBA: (A) Hydrogen exchange in a tetrad. (B) Peak intensities in inversion recovery experiments as a function of mixing time of TBA at 298 K. (C) *k*_ex_ in the absence and presence of 5% thrombin at 298 and 308 K. (D) Temperature dependence of averaged *k*_ex_ in the absence and presence of 5% thrombin, respectively. (E) Exchange rates of TBA at 308 K mapped on 148D.pdb [7]. Buffer conditions used for the measurements (high salt, low catalyst concentration, 137 mM NaCl, 2.7 mM KCl, 10 mM KP_i_, pH 7.4) fall within the conditions required to observe hydrogen exchange under EX_2_ conditions [31].

A deeper understanding of the differential effects on thermal stability between the 3′‐terminal versus the 5′‐terminal variants require investigation at single nucleotide resolution rather than the global melting point in the presence and absence of thrombin. Investigation of the interaction of the different G4s with thrombin by ^1^H NMR spectroscopy is hampered by severe line broadening even at substoichiometric concentrations, making it impossible to separate an appropriate number of imino signals sufficiently to allow measurement over a range of temperatures ( Figure S1). It is, however, possible to probe the changes in G4 stability by hydrogen exchange measurements at a low concentration of thrombin (5%). The measurement of hydrogen exchange [30] monitors the rate at which exchangeable protons, such as those involved in hydrogen bonds within G‐quartets, are replaced by solvent protons (Figure 5A) and the addition of thrombin monitors the differential effect on individual hydrogen bonds, e.g. due to partial protection or deprotection against exchange. For exchange to occur, transient base pair opening must take place (dissociation), exposing the protons to the solvent. In a subsequent step, the proton is exchanged. Slower exchange rates (*k*_ex_) indicate stronger hydrogen bonds and higher structural stability, as base pair opening events are less frequent or energetically unfavorable. Conversely, faster exchange rates suggest weakened interactions or destabilization, as the hydrogen bonds are more prone to disruption.

Figure 5B shows the results of the inversion recovery experiment to measure *k*_ex_ for TBA. Relatively uniform curves are observed, indicating a consistent exchange behavior across the tetrads. Resulting exchange rates for TBA in the absence and presence of thrombin at 298 and 308 K are shown in panel C. Rates range from 0.43 to 0.7 s^−1^ at 298 K in TBA in the absence of thrombin.

These observed rates are low compared to previously measured values in non‐G4 DNA containing Watson–Crick base pairs at similar phosphate catalyst concentrations, which range from 2 to 24 s^−1^ at 298 K [32, 33]. However, the data are in agreement with previous data reporting hydrogen exchange rates below 1 s^−1^ for a telomeric DNA G4 using a different catalyst [34]. Thrombin binding at 298 K leads to an increase in the measured exchange rates reflecting the contribution from cross relaxation due to slower tumbling of the complex at 298 K [35].

The contribution from cross relaxation is also seen in the increase of *k*_ex_ as the temperature is lowered from 298 K, while the individual exchange rates increase (Figure 5D) due to loosening of the hydrogen bonds at temperatures above 298 K. The addition of thrombin leads to a stabilization of hydrogen bridges, particularly near the melting temperature: *k*_ex_ are lower in the presence of thrombin than in the absence for temperatures above 313 K. Both in the presence and absence of thrombin, exchange rates are on average lower for the remote tetrad compared to the rates in the terminal tetrad for temperatures > 308 K, which is close to physiological temperature. Differences within a tetrad are larger than between tetrads. Figure 5E shows the individual *k*_ex_ rates at 308 K mapped onto the respective hydrogen atoms within the structural model of TBA (148d.pdb). While rates in the remote tetrad are rather uniform, variations are larger in the terminal tetrad. The fastest exchanging proton is located at G1, positioned between strands S1 (G1‐G2) and S2 (G5‐G6), whereas the smallest *k*_ex_ is measured for G15. These differences in exchange rates indicate that the strongest interaction occurs between strands S4 (G14‐G15) and S1, contributing to the overall stabilization of the G4 structure.

**FIGURE 6:**
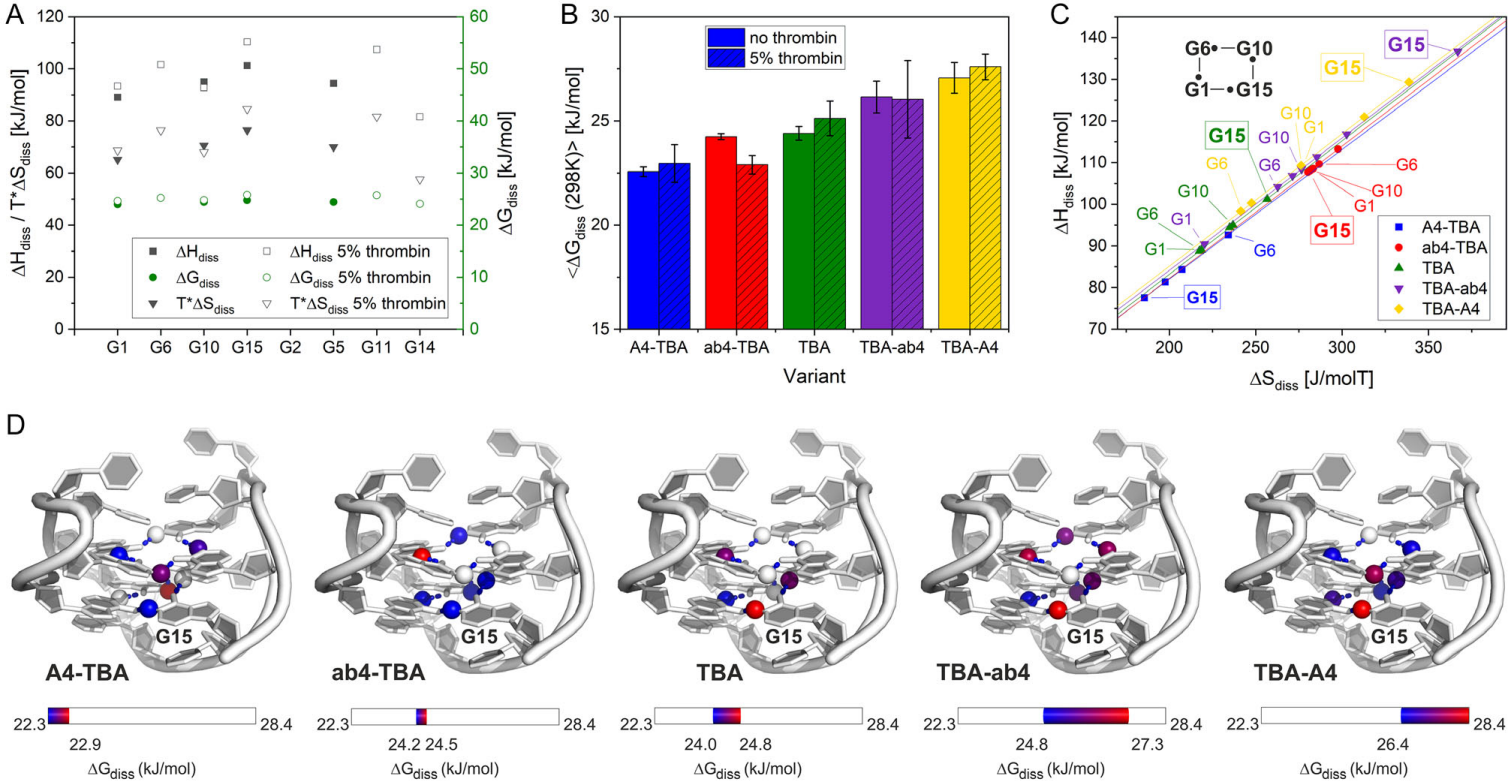
(A) Δ*H*_diss_, TΔS_diss_, and Δ*G*_diss_ of base opening in TBA alone and in the presence of 5% thrombin at 298 K. (B) Averaged Δ*G*_diss_ at 298 K of the variants in the presence (5%) and absence of thrombin. (C) Entropy Enthalpy Compensation plot of the variants in the absence of thrombin. Tedrad architecture of the terminal tedrad is shown, with black balls indicating the position of the observed hydrogen. Values of the terminal are annotated. (D) Δ*G*_diss_ of base opening for the TBA variants in the absence of thrombin mapped on 148D.pdb [7].

Additional insight can be obtained from the determination of enthalpy (Δ*H*_diss_), entropy (Δ*S*_diss_) and free energy (Δ*G*_diss_) of base pair dissociation, which can be derived from the temperature‐dependent measurement of *k*_ex_ [35, 36]. In RNA, the very favorable chemical shift dispersion allows for a nearly complete characteristic of each base pair, as shown previously [35, 37]. In noncanonical DNA G4s, however, the chemical shift dispersion is limited [19], especially within the temperature range of 40K needed to derive Δ*G*_diss_ from *k*_ex_ measurements. Therefore, the completeness of individual *k*_ex_ rates is better than for Δ*H*_diss_, Δ*S*_diss_, and Δ*G*_diss_, even if all available field strengths (600 MHz–1.2 GHz) are exploited. Figure 6A shows Δ*S*_diss_, Δ*H*_diss_, and Δ*G*_diss_ of TBA in the absence and presence of 5% thrombin. The obtained values are relatively uniform along the sequence with Δ*H*_diss_ ranging from 89.1 to 101.3 kJ/mol, Δ*S*_diss_ ranging from 219 to 257 J/(molK) and Δ*G*_diss_ varying between 24.0 and 24.8 kJ/mol (at 298 K) for TBA alone. The values for Δ*H*_diss_ and Δ*S*_diss_ fall in the range of values typically observed in DNA using the same method [32, 33, 38] (Steinert Δ*H*_diss_ 23–203 kJ/mol, ΔS_diss_ −90–593 J/molT, Churcher Δ*H*_diss_ 70–210 kJ/mol, Δ*S*_diss_ 0–570 J/molT, Chen Δ*H*_diss_ 71–117 kJ/mol). However, the variation of the individual dissociation free energies observed for G4s is substantially lower than the variation observed in canonical duplex DNA structures [32, 33, 38] stabilized by Watson–Crick base pairs. In the duplexes [32], very low Δ*H*_diss_ 23–60 kJ/mol) values are observed at the termini, and higher values are observed for central base pairs. Free enthalpy values up to 210 kJ/mol are observed for GC base pairs, which are stabilized by three hydrogen bonds, while values between 70 and 150 kJ/mol are observed for most AT base pairs stabilized by two hydrogen bonds [32, 33, 38]. The values observed here fall within the range of AT base pairs, suggesting that stabilities in the G tetrads are primarly determined by the two hydrogen bonds that are needed to break during base opening (see also Figure 5A). Entropy and enthalpy significantly compensate each other, resulting in relatively low uniform values for Δ*G*_diss_ around 24 kJ/mol at 298 K, which is again within values (between 13 and 76 kJ/mol) [32, 33] observed in DNAs containing Watson–Crick base pairs. Addition of thrombin leads to an increase in Δ*H*_diss_ values of around 5%. However, since Δ*S*_diss_ values also increase, the stabilizing effects are compensated, leading to a minor increase in Δ*G*_diss_ values of base opening.

Averaged Δ*G*_diss_ of all variants is compared in Figure 6B. Δ*G*_diss_ of the more active variants (TBA‐A4 and TBA‐ab4) are higher than observed in TBA, while lower values are observed for the less active variants (A4‐TBA, ab4‐TBA) (Figure 6B). These data suggest that lengthening at the 5′ decreases stability on the level of the base pairs, while addition of adenosines or abasic moieties at the 3′ increases this stability in line with the observation of the melting studies. In the presence of 5% thrombin Δ*G*_diss_ increases for all G4s except for ab4‐TBA.

Entropy and enthalpy are correlated linearly in all variants (Figure 6C), a phenomenon that previously has been observed in the melting of helical nucleic acid structures [32, 35, 38], chemical reactions and unfolding of proteins [39]. The slope of this correlation yields the compensation temperature *T*_C_ at which all Δ*G*_diss_ values have the same value, given by the interception with the *y*‐axis [35, 39]. While folded structures are stabilized by interactions with the solvent below *T*_C_, interactions with the solvent have no net stabilizing effect on the structure at *T*_C_. Due to the small spread of the observed data for a given variant particularly for ab4‐TBA, only a coarse determination of *T*_C_ is possible in this study, however it should be noted that the observed *T*_C_ values are close to the melting temperatures measured by CD with associated Δ*G*_diss_ values at *T*_C_ around 20.0 kJ ± 1.2 kJ (Figure S4).

Although the thermodynamic parameters of the G4 appear relatively uniform, a closer examination reveals key differences. Specifically, G15 exhibits the highest Δ*H*_diss_ and Δ*S*_diss_ values in TBA, TBA‐ab4, and TBA‐A4 (Figure 6C), indicating the strongest stabilization of the structure by the hydrogen bond at this position. In contrast, G15 shows the lowest Δ*H*_diss_ and Δ*S*_diss_ of the variant in A4‐TBA and ab4‐TBA, which is also reflected in the Δ*G*_diss_ values. Addition of nucleotides to the 5′ at G1 thus weakens the hydrogen bonding interactions between G15 and G1. Mapping Δ*G*_diss_ onto the hydrogen atoms of TBA (Figure 6E) highlights a stability hotspot at G15 in TBA and its more active variants, aligning with the exchange rate (*k*_ex_) observations. This interaction, located between strand S1 and strand S4, plays a crucial role in maintaining the overall structural integrity of the G4. However, in ab4‐TBA and A4‐TBA, the stability profile is markedly different, with G15 exhibiting the lowest Δ*G*_diss_ values. Furthermore, in these variants, all values in the terminal tetrad are, in general, lower, suggesting a less stable interaction network at the ends of the structure. The stabilization in the terminal tetrad is also observed in the scarce data due to more overlap in the presence of thrombin (supplementary Tables S6‐S8, supplementary Figure S5). We therefore propose that terminal additions modulate the stability of the hydrogen bonds, which translates into modulation of the stability of the whole G4. The findings provide deeper insight into the structural dynamics of the G4, emphasizing the role of specific interactions in stabilizing the fold, while different to observations in duplexes thermodynamic parameters are remarkably uniform over the structure.

## 3 Conclusion

**FIGURE 7:**
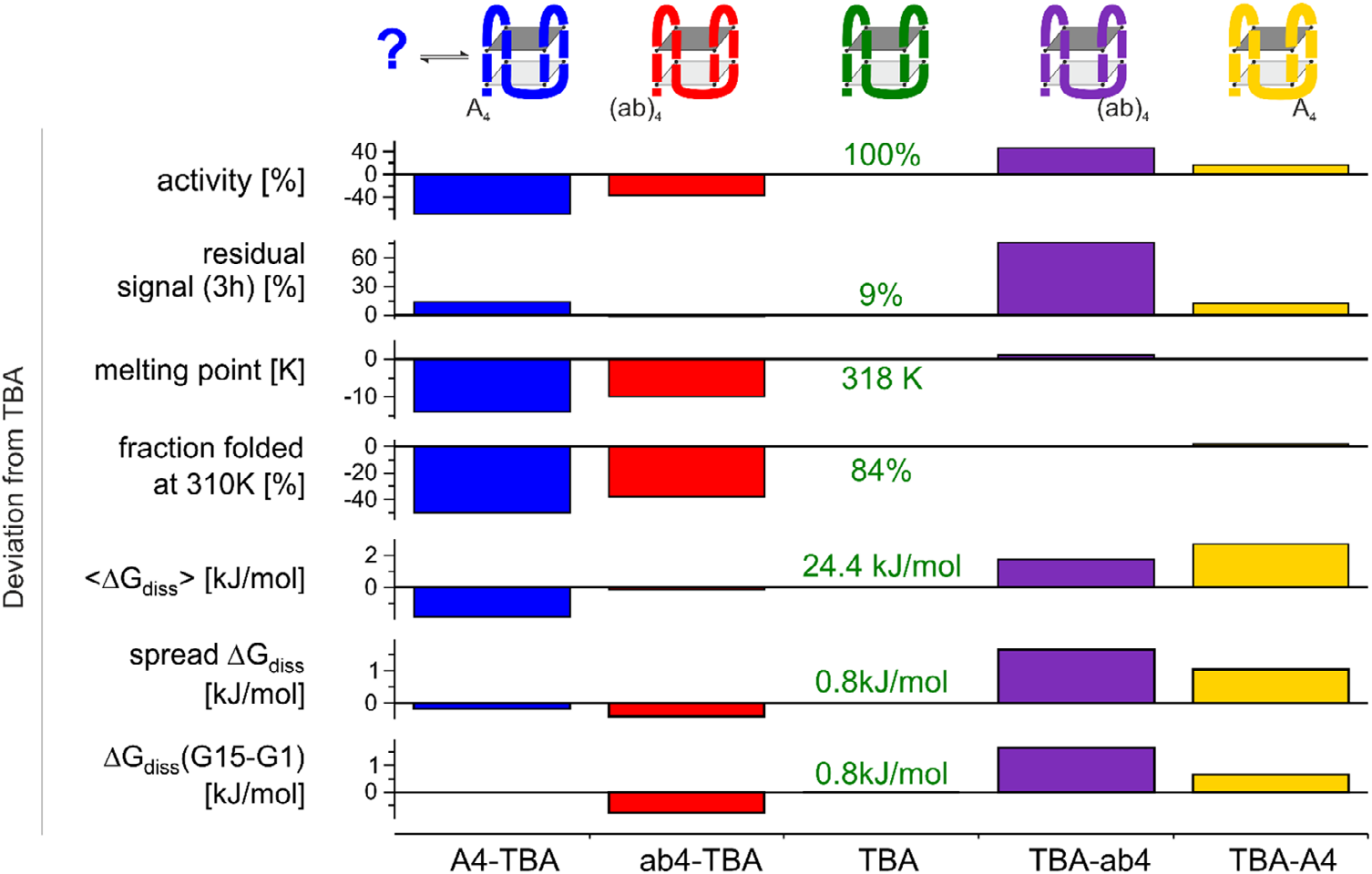
Comparison of variants investigated in this study. Value for TBA is given in the middle, while deviations from these values for the tested variants are given as bars. Activity: from previous anticlotting experiments [18]; residual signal (3h): residual signal intensity of CD signal at 295 nm after incubation at 310 K in FBS (see Figure 3); melting point: from CD melting measurements; fraction folded: remaining CD signal at 295 nm at 310 K based on signal intensity at 278 K (see Figure 4). <Δ*G*_diss_>: averaged Δ*G*_diss_: spread Δ*G*_diss:_ highest ‐ lowest Δ*G*_diss_, Δ*G*_diss_(G15‐G1)‐ difference between Δ*G*_diss_ of G15 and G1 (see Figure 6). Schematic structures of the variants are given in the upper line based on NMR and CD measurements (Figure 2, Figure S1).

In this study, we determined thermal and chemical stability against nuclease digestions by CD and individual base pair stability by hydrogen exchange NMR measurements to investigate the impact of terminal abasic and adenosine modifications of TBA variants, linking these findings to their coagulation activity (see Figure 7). All variants maintain antiparallel G4 structures, sharing a conserved core architecture including two G‐quartets; however, the least active variant populates a minor conformation as evidenced in NMR and CD measurements in FBS.

Hydrogen exchange experiments of the G4s reveal relatively uniform dissociation rates across Hoogsteen interactions in the G4s, this contrasts with terminal base pairs in helical nucleic acid structures which exhibit significant lower stability than central base pairs. Accordingly, relatively uniform Δ*H*_diss_, Δ*S*_diss_, and Δ*G*_diss_ values are observed throughout the investigated sequences, indicating the high interdependence of all Hoogsteen and stacking interactions within the G4 for stabilizing the fold. Despite this overall uniformity, subtle differences in basepair thermodynamics reveal a hotspot for the stabilization of the TBA G4: Δ*H*_diss_ and Δ*S*_diss_ are highest for G15 in the active variants (TBA‐A4, TBA‐ab4, and TBA) reporting on the stabilization of the hydrogen bond between G15 and G1, while Δ*H*_diss_ and Δ*S*_diss_ are lowest for G15 in the variants with decreased coagulation activity such as ab4‐TBA and A4‐TBA. These less active variants share lower melting temperatures and smaller populations of folded conformation at 310 K, as well as decreased Δ*G*_diss_ values for basepair opening, a narrower distribution of Δ*G*_diss_ values within the G4 and a small difference between Δ*G*_diss_ values in the terminal tetrad (G15 versus G1). Interestingly, the stability against digestion by nucleases in FBS is in the same time range as observed for TBA.

In contrast, the more active variants share similar melting temperatures and folded‐state populations at 310 K as TBA but show increased Δ*G*_diss_ of basepair opening, a broader spread of Δ*G*_diss_ values in the G4 and a particular hotspot for stability based on the difference of Δ*G*_diss_ values of G15 and G1. The most active variant TBA‐ab4 exhibits significant nuclease resistance in FBS, while all other variants decay to less than 25% or even 10% within 3h. This suggests that 3′‐terminal abasic sites enhance nuclease resistance compared to adenine (A) residues in this context.

Overall, the findings indicate that both global stability and localized thermodynamic features within the G4, particularly at G15, together with the nature of terminal modifications, govern the anticoagulant activity and nuclease resistance of TBA variants.

## Supporting Information

The authors have cited additional references within the Supporting Information [40]. Additional supporting information can be found online in the Supporting Information section.

## Funding

This work was supported by Deutsche Forschungsgemeinschaft (Grant 531012774) and Hessisches Ministerium für Wissenschaft und Kunst Grant (BMRZ).

## Conflicts of Interest

The authors declare no conflicts of interest.

## Supporting information

Supplementary Material

## Data Availability

NMR raw data for assignment and hydrogen exchange as well as raw data of CD melting curves have been deposited in GUDe https://gude.uni‐frankfurt.de/handle/gude/656with DOI: https://doi.org/10.25716/gude.1rvp‐c027. NMR Assignments are deposited in the BMRB with the IDs ab4‐TBA 53334, A4‐TBA 53335, TBA‐A4 53337 and TBA‐ab4 53336.

## References

[1] D. M. Monroe and M. Hoffman , “What Does It Take to Make the Perfect Clot?,” Arteriosclerosis, Thrombosis, and Vascular Biology 26 (2006): 41–48. 16254201 10.1161/01.ATV.0000193624.28251.83

[2] R. De Cristofaro , E. De Candia , and I. Medicine , “Thrombin Domains: Structure, Function and Interaction with Platelet Receptors,” Journal of Thrombosis and Thrombolysis 15 (2003): 151–163. 14739624 10.1023/B:THRO.0000011370.80989.7b

[3] S. Pervaiz Butt , V. Kakar , A. Kumar , et al., “Heparin Resistance Management during Cardiac Surgery: A Literature Review and Future Directions,” The Journal of ExtraCorporeal Technology 56 (2024): 136–144. 10.1051/ject/2024015 PMC11415039 39303137

[4] R. L. Bick and E. P. Frenkel , “Clinical Aspects of Heparin‐Induced Thrombocytopenia and Thrombosis and Other Side Effects of Heparin Therapy,” Clinical and Applied Thrombosis/Hemostasis 5 (1999): S7. 10726030 10.1177/10760296990050s103

[5] Y. Ning , J. Hu , and F. Lu , “Aptamers Used for Biosensors and Targeted Therapy,” Biomedicine & Pharmacotherapy 132 (2020): 110902. 33096353 10.1016/j.biopha.2020.110902 PMC7574901

[6] L. C. Bock , L. C. Griffin , J. A. Latham , E. H. Vermaas , and J. J. Toole , “Selection of Single‐Stranded DNA Molecules that Bind and Inhibit Human Thrombin,” Nature 355 (1992): 564–566. 1741036 10.1038/355564a0

[7] P. Schultze , R. F. Macaya , and J. Feigon , “Three‐Dimensional Solution Structure of the Thrombin‐Binding DNA Aptamer d(GGTTGGTGTGGTTGG),” Journal of Molecular Biology 235 (1994): 1532–1547. 8107090 10.1006/jmbi.1994.1105

[8] I. R. Krauss , A. Merlino , C. Giancola , A. Randazzo , L. Mazzarella , and F. Sica , “Thrombin–aptamer Recognition: A Revealed Ambiguity,” Nucleic Acids Research 39 (2011): 7858–7867. 21715374 10.1093/nar/gkr522 PMC3177225

[9] I. Russo Krauss , A. Merlino , A. Randazzo , E. Novellino , L. Mazzarella , and F. Sica , “High‐Resolution Structures of Two Complexes between Thrombin and Thrombin‐Binding Aptamer Shed Light on the Role of Cations in the Aptamer Inhibitory Activity,” Nucleic Acids Research 40 (2012): 8119. 22669903 10.1093/nar/gks512 PMC3439905

[10] K. Padmanabhan , K. P. Padmanabhan , J. D. Ferrara , J. E. Sadler , and A. Tulinsky , “The Structure of Alpha‐Thrombin Inhibited by a 15‐Mer Single‐Stranded DNA Aptamer.,” The Journal of Biological Chemistry 268 (1993): 17651–17654. 8102368 10.2210/pdb1hut/pdb

[11] J. A. Kelly , J. Feigon , and T. O. Yeates , “Reconciliation of the X‐Ray and NMR Structures of the Thrombin‐Binding Aptamer d(GGTTGGTGTGGTTGG),” Journal of Molecular Biology 256 (1996): 417. 8604127 10.1006/jmbi.1996.0097

[12] K. Padmanabhan and A. Tulinsky , “An Ambiguous Structure of a DNA 15‐Mer Thrombin Complex,” Acta Crystallographica Section D Biological Crystallography 52 (1996): 272–282. 15299700 10.1107/S0907444995013977

[13] Y. Kasahara , S. Kitadume , K. Morihiro , et al., “Effect of 3′‐End Capping of Aptamer with Various 2′,4′‐Bridged Nucleotides: Enzymatic Post‐Modification toward a Practical use of Polyclonal Aptamers,” Bioorganic & Medicinal Chemistry Letters 20 (2010): 1626–1629. 20153191 10.1016/j.bmcl.2010.01.028

[14] B. Cai , X. Yang , L. Sun , et al., “Stability and Bioactivity of Thrombin Binding Aptamers Modified with d‐/l‐Isothymidine in the Loop Regions,” Organic & Biomolecular Chemistry 12 (2014): 8866–8876. 25264858 10.1039/c4ob01525h

[15] C. Riccardi , E. Napolitano , C. Platella , D. Musumeci , and D. Montesarchio , “G‐Quadruplex‐Based Aptamers Targeting Human Thrombin: Discovery, Chemical Modifications and Antithrombotic Effects,” Pharmacology & Therapeutics 217 (2021): 107649. 32777331 10.1016/j.pharmthera.2020.107649

[16] K. Suckling , “Discontinued Drugs in 2005: Cardiovascular Drugs,” Expert Opinion on Investigational Drugs 15 (2006): 1299–1308. 17040192 10.1517/13543784.15.11.1299

[17] A. Schwienhorst , “Direct Thrombin Inhibitors – a Survey of Recent Developments,” Cellular and Molecular Life Sciences 63 (2006): 2773–2791. 17103113 10.1007/s00018-006-6219-z PMC11135997

[18] M. C. R. Buff , F. Schäfer , B. Wulffen , et al., “Dependence of Aptamer Activity on Opposed Terminal Extensions: Improvement of Light‐Regulation Efficiency,” Nucleic Acids Research 38 (2009): 2111–2118. 20007153 10.1093/nar/gkp1148 PMC2847219

[19] M. Adrian , B. Heddi , and A. T. Phan , “NMR Spectroscopy of G‐Quadruplexes,” Methods 57 (2012): 11–24. 22633887 10.1016/j.ymeth.2012.05.003

[20] R. F. Macaya , P. Schultze , F. W. Smith , J. A. Roe , and J. Feigon , “Thrombin‐Binding DNA Aptamer Forms a Unimolecular Quadruplex Structure in Solution,” Proceedings of the National Academy of Sciences of the United States of America 90 (1993): 3745. 8475124 10.1073/pnas.90.8.3745 PMC46378

[21] A. Virgilio , D. Benigno , C. Aliberti , et al., “Improving the Biological Properties of Thrombin‐Binding Aptamer by Incorporation of 8‐Bromo‐2'‐Deoxyguanosine and 2'‐Substituted RNA Analogues,” International Jounal of Molecular Sciences 24 (2023): 15529. 10.3390/ijms242115529 PMC10647374 37958511

[22] S. Uehara , N. Shimada , Y. Takeda , et al., “3′ Poly(dA)‐Tailed Thrombin DNA Aptamer to Increase DNase‐Resistance and Clotting Inhibitory Activity,” Bulletin of the Chemical Society of Japan 81 (2008): 1485–1491.

[23] A. De Rache , I. Kejnovská , M. Vorlíčková , and C. Buess‐Herman , “Elongated Thrombin Binding Aptamer: A G‐Quadruplex Cation‐Sensitive Conformational Switch,” Chemistry a European Journal 18 (2012): 4392– 4400. 22362492 10.1002/chem.201103381

[24] V. B. Tsvetkov , A. M. Varizhuk , G. E. Pozmogova , I. P. Smirnov , N. A. Kolganova , and E. N. Timofeev , “A Universal Base in a Specific Role: Tuning up a Thrombin Aptamer with 5‐Nitroindole,” Scientific Reports 5 (2015): 1–11.

[25] C. Riccardi , I. Russo Krauss , D. Musumeci , et al., “Fluorescent Thrombin Binding Aptamer‐Tagged Nanoparticles for an Efficient and Reversible Control of Thrombin Activity,” ACS Applied Materials & Interfaces 9 (2017): 35574–35587. 28849915 10.1021/acsami.7b11195

[26] C. Riccardi , A. Meyer , J. J. Vasseur , et al., “Fine‐Tuning the Properties of the Thrombin Binding Aptamer through Cyclization: Effect of the 5′‐3′ Connecting Linker on the Aptamer Stability and Anticoagulant Activity,” Bioorganic Chemistry 94 (2020): 103379. 31699393 10.1016/j.bioorg.2019.103379

[27] C. Riccardi , A. Meyer , J. J. Vasseur , et al., “Stability Is Not Everything: The Case of the Cyclisation of a Thrombin‐Binding Aptamer,” ChemBioChem 20 (2019): 1789. 30860635 10.1002/cbic.201900045

[28] K. Ikebukuro , Y. Okumura , K. Sumikura , and I. Karube , “A Novel Method of Screening Thrombin‐Inhibiting DNA Aptamers using an Evolution‐Mimicking Algorithm,” Nucleic Acids Rsearch 33 (2005): 1–7. 10.1093/nar/gni108 PMC1174901 16002787

[29] E. G. Zavyalova , V. A. Legatova , R. S. Alieva , et al., “Putative Mechanisms Underlying High Inhibitory Activities of Bimodular DNA Aptamers to Thrombin,” Biomolecules9 (2019): 1–15. 10.3390/biom9020041 PMC6406280 30682825

[30] M. Guéron and J. L. Leroy , “Studies of Base Pair Kinetics by NMR Measurement of Proton Exchange,” Methods in Enzymology 261 (1995): 383–413. 8569504 10.1016/s0076-6879(95)61018-9

[31] S. W. Englander , T. R. Sosnick , J. J. Englander , and L. Mayne , “Mechanisms and Uses of Hydrogen Exchange,” Current Opinion in Structural Biology 6 (1996): 18–23. 8696968 10.1016/s0959-440x(96)80090-x PMC3412065

[32] H. S. Steinert , J. Rinnenthal , and H. Schwalbe , “Individual Basepair Stability of DNA and RNA Studied by NMR‐Detected Solvent Exchange,” Biophysical Journal 102 (2012): 2564–2574. 22713572 10.1016/j.bpj.2012.03.074 PMC3368144

[33] Z. R. Churcher , D. Garaev , H. N. Hunter , and P. E. Johnson , “Reduction in Dynamics of Base Pair Opening upon Ligand Binding by the Cocaine‐Binding Aptamer,” Biophysical Journal 119 (2020): 1147–1156. 32882188 10.1016/j.bpj.2020.08.012 PMC7499103

[34] Y. Yamaoki , T. Nagata , K. Kondo , T. Sakamoto , S. Takami , and M. Katahira , “Shedding Light on the Base‐Pair Opening Dynamics of Nucleic Acids in Living Human Cells,” Nature Communications 13 (2022): 7143. 10.1038/s41467-022-34822-4 PMC9708698 36446768

[35] J. Rinnenthal , B. Klinkert , F. Narberhaus , and H. Schwalbe , “Direct Observation of the Temperature‐Induced Melting Process of the Salmonella fourU RNA Thermometer at Base‐Pair Resolution,” Nucleic Acids Research 38 (2010): 3834–3847. 20211842 10.1093/nar/gkq124 PMC2887971

[36] Y. Bai , J. J. Englander , L. Mayne , J. S. Milne , and S. W. Englander , “Thermodynamic Parameters from Hydrogen Exchange Measurements,” Methods in Enzymology 259 (1995): 344–356. 8538461 10.1016/0076-6879(95)59051-x

[37] D. Wagner , J. Rinnenthal , F. Narberhaus , and H. Schwalbe , “Mechanistic Insights into Temperature‐Dependent Regulation of the Simple Cyanobacterial hsp17 RNA Thermometer at Base‐Pair Resolution,” Nucleic Acids Research 43 (2015): 5572–5585. 25940621 10.1093/nar/gkv414 PMC4477652

[38] C. Chen and I. M. Russu , “Sequence‐Dependence of the Energetics of Opening of AT Basepairs in DNA,” Biophysical Journal 87 (2004): 2545–2551. 15454449 10.1529/biophysj.104.045179 PMC1304673

[39] R. Lumry and S. Rajender , “Enthalpy‐entropy Compensation Phenomena in Water Solutions of Proteins and Small Molecules: A Ubiquitous Property of Water,” Biopolymers 9 (1970): 1557–1559. 10.1002/bip.1970.360091002 4918636

[40] W. Lee , M. Tonelli , and J. L. Markley , “NMRFAM‐SPARKY: Enhanced Software for Biomolecular NMR Spectroscopy,” Bioinformatics 31 (2015): 1325–1327. 25505092 10.1093/bioinformatics/btu830 PMC4393527

