# Supplementary material for: Dissecting the Chemical and Thermal Stabilities of Tetrads in G‐Quadruplexes to Derive a Structure‐Activity Relation for a Thrombin‐Binding DNA G‐Quadruplex Aptamer
Source: Chembiochem. 2026 Apr 21;27(8):e202500743. doi: 10.1002/cbic.202500743 (PMC13097084; doi:10.1002/cbic.202500743)
Supplement: Supplementary file 1 — Supplementary Material [file CBIC-27-e202500743-s001.pdf]

Supplementary Material

Dissecting the Chemical and Thermal Stabilities of Tetrads in G-Quadruplexes to Derive a Structure-Activity Relation for a Thrombin-Binding DNA G-Quadruplex Aptamer

Julia Wirmer-Bartoschek,[a] Jan-Peter Ferner, [a] Alexander Heckel,[b] and Harald Schwalbe*[a]

[a] Dr. J. Wirmer-Bartoschek, Dr. J.-P. Ferner, Prof. Dr. H.Schwalbe
Institute for Organic Chemistry and Chemical Biology, Center for Biomolecular Magnetic Resonance (BMRZ)
Johann Wolfgang Goethe University
Max von Laue Str. 7, 60438 Frankfurt am Main (Germany)
E-mail: schwalbe@nmr.uni-frankfurt.de

[b] Prof. Dr. A. Heckel
Institute for Organic Chemistry and Chemical Biology
Johann Wolfgang Goethe University
Max von Laue Str. 7, 60438 Frankfurt am Main (Germany)

Methods:

All measurements but the degradation measurements were conducted in 137mM NaCl, 2.7mM KCl, 10mM KPi, pH 7.4. CD samples had a concentration of 7.5µM, NMR samples for assignment 1mM for TBA-A4 and TBA-ab4, 1.5mM for ab4-TBA and 2mM for A4-TBA. NMR samples for hydrogen exchange measurements contained 2mM or 0.3mM of the respective TBA variant. CD measurements were performed on a JASCO J-810S CD-spectrometer. Measurements in buffer were performed using bandwidth 1nm, D.I.T. 1s. Melting was monitored at a constant wavelength of 294nm, ramping from 5-95°C at 0.5°C/min.

Degradation measurements were performed following Virgilio et al.[23] Samples contained 30µM of the TBA variant in 10% NMR buffer (see above), 50% fetal bovine serum (FBS) and 50% DMEM (Dulbecco's modified eagles medium, high glucose pH 7-7.4. Samples were obtained by lyophilizing 22ul of 300µM TBA variant in NMR buffer and resuspending in 220ul DMEM:FBS 1:1 at 4°C. Measurements were performed at 37°C- 1st spectrum was recorded 100 seconds after transferring to 37°C using bandwidth 2nm, D.I.T. 4s.

Assignment was performed at 278K using ^{15}N HSQC, ^{1}H,^{1}H NOESY and ^{1}H,^{1}H TOCSY experiments from the Bruker library. Spectra were acquired at Bruker instruments ranging from 600 to 950MHz.

Spectra were processed in TOPSPIN and analyzed using NMRFAM-SPARKY [40].

Hydrogen exchange measurements were performed at Bruker 600MHz spectrometer using inversion recovery experiments following Rinnenthal et al [35]. Data were fitted using Origin Pro to the following formular:

$(t)(0)-1=-2k_{ex}\exp(-R_{ln}t)-\exp(-R_{lw}t) / (-R_{ln}+R_{lw})$

with R_{1w}= R_1 water – global fit per temperature, R_{1n}=R_1 of nucleotide, t= delay, k_{ex} apparent exchange rate

Fitting of H_{diss} and S_{diss}:

Severe peak overlap of imino peaks is present in the construct, with varying degrees for the different temperatures. Therefore exchange rates for overlapping peaks were not included in the fitting procedure, resulting in fewer points for fitting and /or in bases that could not be fitted at all.

ΔH_{Diss} and ΔS_{Diss} were determined using the following fitting function:

$$k_{ex} = \frac{\frac{k_b T}{h} \cdot \exp\left(-\frac{\Delta H_{TR} - T\Delta S_{TR}}{RT}\right)}{1+\exp\left(\frac{\Delta H_{Diss} - T\Delta S_{Diss}}{RT}\right)} + d(T) \qquad (1)$$

where k_B is the Boltzmann constant, h is the Planck constant, and R is the gas constant. ΔH_{TR} and ΔS_{TR} are parameters representing the transition state of the imino proton exchange between water and nucleobases in the open state. These values were adopted from Rinnenthal et al. [3]

$$\Delta H_{TR} = 38580 \frac{J}{mol} \qquad \Delta S_{TR} = -50 \frac{J}{mol \cdot K}$$

ΔH_{Diss} and ΔS_{Diss} describe the equilibrium reaction of base opening and closing.

Measured k_{ex} contain contributions from crossrelaxation during the recovery delay. This can be described by

$$d(T) = d(283K) \cdot \left[a \cdot \exp\left(-b \cdot (T - 273.1K)\right) + c \cdot \exp\left(-f \cdot (T - 273.1K)\right)\right]$$

with a=0.8230 b=0.0505 c=0.5725 f=0.0129 as determined by Rinnenthal.

References:

[21] A. Virgilio, D. Benigno, C. Aliberti, V. Vellecco, V. Esposito, A. Galeone, M. Bucci, "Improving the Biological Properties of Thrombin-Binding Aptamer by Incorporation of 8-Bromo-2'-Deoxyguanosine and 2'-Substituted RNA Analogues," *International Jounal of Molecular Sciences* 24 (2023): 15529.

[35] J. Rinnenthal, B. Klinkert, F. Narberhaus, and H. Schwalbe, "Direct Observation of the Temperature-Induced Melting Process of the Salmonella fourU RNA Thermometer at Base-Pair Resolution," *Nucleic Acids Research* 38 (2010): 3834–3847.

[40] W. Lee, M. Tonelli, and J. L. Markley, "NMRFAM-SPARKY: Enhanced Software for Biomolecular NMR Spectroscopy," *Bioinformatics* 31 (2015): 1325–1327.

Open Science:

NMR raw data for assignment and hydrogen exchange as well as raw data of CD melting curves have been deposited in GUDe https://gude.uni-frankfurt.de/handle/gude/656

with DOI:

https://doi.org/10.25716/gude.1rvp-c027

Assignments are deposited in the BMRB with the IDs ab4-TBA 53334, A4-TBA 53335, TBA-A4 53337 and TBA-ab4 53336.

Supplementary Figures:

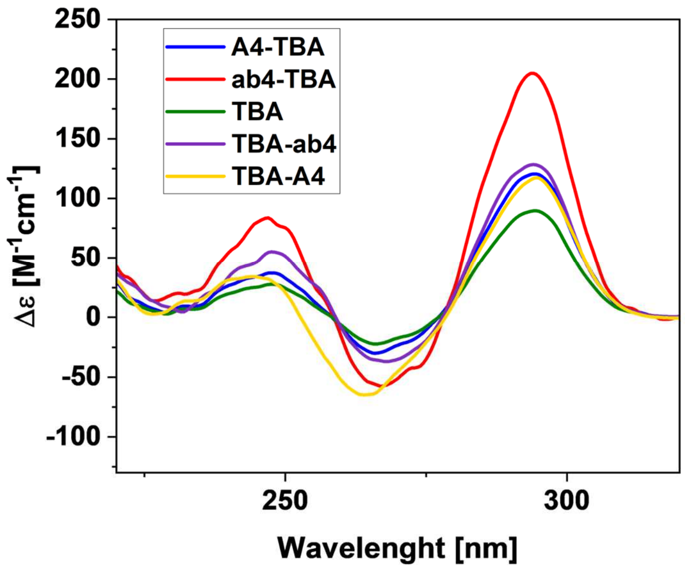

Supplementary Figure S1: Static CD spectra of TBA and its variants at 293 K

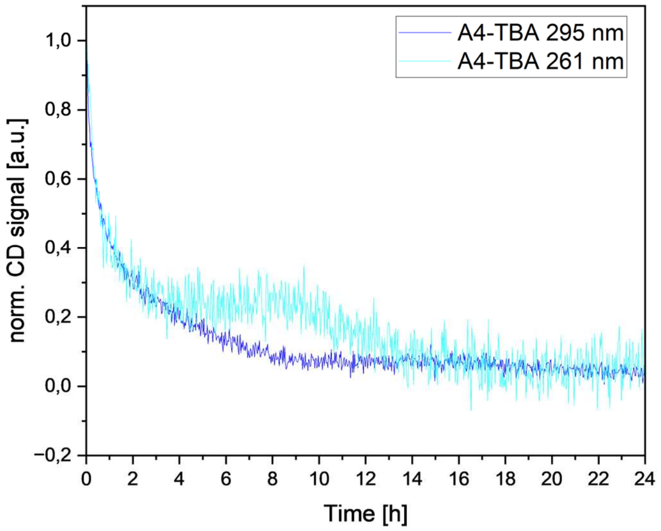

Supplementary Figure S2: Time dependent decay of the CD signals of a4-TBA at 295 and 261 nm at 310 K in FBS:DMEM 1:1

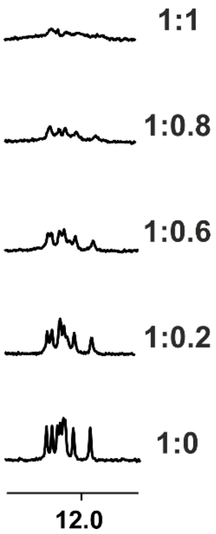

Supplementary Figure S3: ^{1}H NMR spectrum of the Iminoregion of the titration of TBA-ab4 with thrombin at 298 K, 0.1 mM TBA-ab4, 137 mM NaCl, 2.7 mM KCl, 10 mM KPi, pH 7.4.

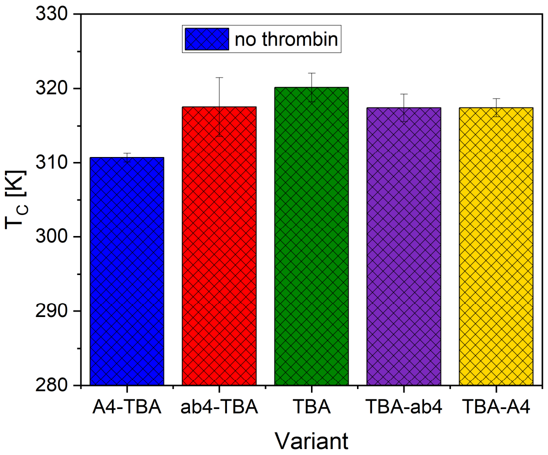

Supplementary Figure S4: Compensation temperatures derived from linear fitting of entropy-enthalpy correlation (see Figure 6 in main manuscript) for the different variants.

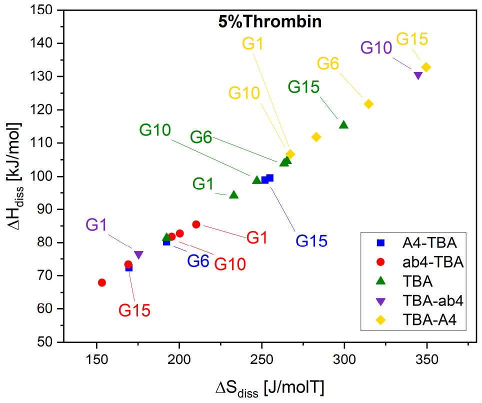

Supplementary Figure S5: Entropy Enthalpy Compensation plot of the variants in the presence of 5% thrombin. Values for the terminal tetrad are annotated.

Supplementary Tables:

Table S1: k_{ex} values of TBA in Hz. kex of overlapping signals were fitted together- resulting k_{ex} are listed for both bases. In the fitting procedure for ΔH_{diss} and ΔS_{diss}, k_{ex} from overlapping signals were not used. Shown k_{ex} are not corrected for crossrelaxation.

	k_{ex} of TBA								
	278K	283K	288K	293K	298K	303K	308K	313K	318K
**G1**	0.86207	0.75865	0.61296	0.57049	0.63328	0.77172	1.32877	2.31619	4.76197
**G1Err**	0.02104	0.01508	0.01246	0.01046	0.01423	0.01325	0.02889	0.03649	0.07847
**G6**	0.95849	0.82186	0.70683	0.60827	0.67532	0.69671	1.31872	2.31374	4.66276
**G6Err**	0.02356	0.01568	0.01386	0.01152	0.0171	0.01398	0.03074	0.03781	0.07787
**G10**	0.85919	0.70173	0.50616	0.49151	0.5582	0.66816	1.20159	2.21534	4.62541
**G10Err**	0.02131	0.01378	0.01089	0.00999	0.01477	0.01285	0.02782	0.03659	0.07837

**G15**	0.64642	0.56657	0.45745	0.38549	0.43281	0.59998	1.06468	2.09305	4.50933
**G15Err**	0.02078	0.01396	0.01146	0.00887	0.01119	0.01094	0.02405	0.03356	0.07514
**G2**	0.80215	0.82186	0.70683	0.60827	0.5793	0.76077	1.22984	2.17284	4.50933
**G2Err**	0.02278	0.01568	0.01386	0.01152	0.01467	0.01402	0.02838	0.03548	0.07514
**G5**	0.85917	0.83895	0.70242	0.63764	0.69758	0.77099	1.22984	2.24058	4.6226
**G5Err**	0.01969	0.01414	0.01239	0.01101	0.01616	0.01408	0.02838	0.03712	0.07807
**G11**	0.8657	0.75865	0.61296	0.52802	0.58317	0.69671	1.2036	2.22018	4.5508
**G11Err**	0.02321	0.01508	0.01246	0.01097	0.01551	0.01398	0.02795	0.03671	0.07776
**G14**	0.90204	0.82186	0.70683	0.60827	0.64814	0.8058	1.15643	2.22018	4.5508
**G14Err**	0.02129	0.01568	0.01386	0.01152	0.01599	0.01512	0.02738	0.03671	0.07776

Table S2: k_{ex} values of TBA in the presence of 5% thrombin in Hz. k_{ex} of overlapping signals were fitted together- resulting k_{ex} are listed for both bases. In the fitting procedure for ΔH_{diss} and ΔS_{diss}, k_{ex} from overlapping signals were not used. Shown k_{ex} are not corrected for crossrelaxation.

k_{ex} of TBA in the presence of 5% thrombin									
	278K	283K	288K	293K	298K	303K	308K	313K	318K
**G1**	1.73842	1.33403	1.11093	0.93662	0.87583	1.05012	1.39363	2.29328	4.33998
**G1Err**	0.03946	0.02741	0.02104	0.01704	0.01689	0.02189	0.02458	0.04231	0.08454
**G6**	1.90653	1.57452	1.3157	1.01153	1.07036	1.07888	1.29738	2.23826	4.30758
**G6Err**	0.04332	0.03246	0.02485	0.01964	0.02193	0.0258	0.02616	0.0445	0.08701
**G10**	1.5362	1.1653	0.96511	0.70819	0.74359	0.86311	1.24424	2.19558	3.94616
**G10Err**	0.03652	0.02589	0.01963	0.0146	0.01584	0.0201	0.02435	0.04318	0.07942
**G15**	1.33832	1.07526	0.83013	0.62113	0.58813	0.71507	1.05056	1.98815	4.05281
**G15Err**	0.03803	0.0283	0.01993	0.01424	0.01358	0.01665	0.01937	0.03768	0.0791
**G2**	1.73842	1.49817	1.24461	1.01153	0.9993	1.05879	1.27963	2.13618	3.99819
**G2Err**	0.03946	0.03226	0.02552	0.01964	0.0202	0.02485	0.02613	0.04355	0.08129
**G5**	1.98052	1.59913	1.39237	1.12248	1.10094	1.11043	1.40062	2.13618	3.99819
**G5Err**	0.04077	0.03013	0.02447	0.01905	0.02075	0.02548	0.02822	0.04355	0.08129
**G11**	2.0202	1.35293	1.11093	0.93662	0.87583	1.07473	1.29738	2.13611	4.10819
**G11Err**	0.04353	0.02914	0.02104	0.01704	0.01689	0.02567	0.02616	0.04424	0.08421
**G14**	1.90653	1.57452	1.3157	1.0508	0.9993	1.05879	1.45883	2.28682	4.09066
**G14Err**	0.04332	0.03246	0.02485	0.01904	0.0202	0.02485	0.0294	0.04645	0.08379

Table S3: ΔH_{diss} values of respective variants without thrombin

	A4-TBA		ab4-TBA		TBA		TBA-ab4		TBA-A4	
	ΔH_{diss} [kJ/mol]	error	ΔH_{diss} [kJ/mol]	error	ΔH_{diss} [kJ/mol]	error	ΔH_{diss} [kJ/mol]	error	ΔH_{diss} [kJ/mol]	error
**G1**			108.58	21.04	89.11	3.52	90.49	13.27	109.29	16.12
**G6**	92.66	4.67	109.65	32.46			104.19	17.14	98.33	19.31
**G10**			108.04	14.77	94.98	5.07	108.47	14.78	109.29	16.12
**G15**	77.58	5.12	107.73	13.96	101.26	7.80	136.66	31.43	129.33	22.57
**G2**	84.37	7.85							120.95	20.53
**G5**			113.23	23.61	94.44	4.54	116.83	18.10	100.32	16.42
**G11**	84.37	7.85	108.39	18.99			106.86	17.98		
**G14**	81.36	8.15					111.38	18.46	100.32	16.42

G2 and G5 of A4-TBA overlap over the whole temperature range

Table S4: ΔS_{diss} values of respective variants without thrombin

	A4-TBA		ab4-TBA		TBA		TBA-ab4		TBA-A4	
	ΔS_{diss} J/molT	error	ΔS_{diss} J/molT	error	ΔS_{diss} J/molT	error	ΔS_{diss} J/molT	error	ΔS_{diss} J/molT	error
**G1**			283.28	56.08	218.54	9.56	220.36	36.18	276.34	42.28
**G6**	234.11	12.57	286.78	86.08			262.77	45.49	241.36	51.63
**G10**			281.40	39.25	236.79	13.62	276.53	38.96	276.34	42.28
**G15**	185.58	14.20	280.31	37.14	256.61	20.99	366.98	79.23	338.70	56.92
**G2**	207.29	21.48							312.83	52.59
**G5**			297.64	62.34	234.99	12.21	302.61	46.80	247.60	43.81
**G11**	207.29	21.48	282.44	50.32			271.30	47.36		
**G14**	197.62	22.41					285.37	48.23	247.60	43.81

G2 and G11 of A4-TBA overlap over the whole temperature range

Table S5: ΔG_{diss} 298K values of respective variants

	**A4-TBA**		**ab4-TBA**		**TBA**		**TBA-ab4**		**TBA-A4**	
	ΔG_{diss} 298K [kJ/mol]	error	ΔG_{diss} 298K [kJ/mol]	error	ΔG_{diss} 298K [kJ/mol]	error	ΔG_{diss} 298K [kJ/mol]	error	ΔG_{diss} 298K [kJ/mol]	error
**G1**			24.16	21.06	23.99	3.53	24.83	13.28	26.94	16.14
**G6**	22.90	4.67	24.19	32.49			25.88	17.16	26.40	19.33
**G10**			24.19	14.78	24.42	5.08	26.07	14.80	26.94	16.14
**G15**	22.27	5.13	24.20	13.97	24.79	7.81	27.30	31.46	28.40	22.59
**G2**	22.60	7.86							27.73	20.55
**G5**			24.54	23.63	24.42	4.55	26.65	18.12	26.54	16.44
**G11**	22.60	7.86	24.22	19.01	0.00	0.00	26.01	18.00		
**G14**	22.47	8.15	0.00	0.00	0.00	0.00	26.34	18.48	26.54	16.44

G2 and G11 of A4-TBA overlap over the whole temperature range

Table S6: ΔH_{diss} values of respective variants in the presence of 5% thrombin

	**A4-TBA + 5% thrombin**		**ab4-TBA + 5% thrombin**		**TBA + 5% thrombin**		**TBA-ab4 + 5% thrombin**		**TBA-A4 + 5% thrombin**	
	ΔH_{diss} kJ/mol	error	ΔH_{diss} kJ/mol	error	ΔH_{diss} kJ/mol	error	ΔH_{diss} kJ/mol	error	ΔH_{diss} kJ/mol	error
**G1**			85.44	2.68	93.35	1.07	84.33	31.71	106.63	27.60
**G6**	80.23	22.08			101.59	4.21			121.75	38.86
**G10**			81.75	14.27	92.78	13.52	123.14	35.30	106.63	27.60
**G15**	98.94	21.20	73.35	11.45	110.39	14.06			132.92	37.13
**G2**	99.57	23.61								
**G5**	72.39	19.31							111.78	35.27
**G11**	99.57	23.61			107.46	29.20				

G2 and G11 of A4-TBA overlap over the whole temperature range, G5 and G14 of A4-TBA and TBA-A4 overlap over the whole temperature range used for fitting

Table S7: ΔS_{diss} values of respective variants in the presence of 5% thrombin

	**A4-TBA + 5% thrombin**		**ab4-TBA + 5% thrombin**		**TBA + 5% thrombin**		**TBA-ab4 + 5% thrombin**		**TBA-A4 + 5% thrombin**	
	ΔS_{diss} J/molT	error	ΔS_{diss} J/molT	error	ΔS_{diss} J/molT	error	ΔS_{diss} J/molT	error	ΔS_{diss} J/molT	error
**G1**			210.33	7.38	230.54	2.87	200.14	86.64	267.24	72.87
**G6**	192.33	62.02			256.24	11.13			314.71	99.51
**G10**			195.47	39.31	228.08	36.46	321.15	89.92	267.24	72.87
**G15**	251.87	57.40	169.19	32.04	283.80	36.68			349.54	93.15
**G2**	254.88	63.79	153.17	64.70						
**G5**	169.53	55.26							282.99	92.18
**G11**	254.88	63.79			274.16	76.56				
**G14**	169.53	55.26	200.34	48.94	193.12	1.31			282.99	92.18

G2 and G11 of A4-TBA overlap over the whole temperature range, G5 and G14 of A4-TBA and TBA-A4 overlap over the whole temperature range used for fitting

Table S8: ΔG_{diss}(298K) values of respective variants in the presence of 5% thrombin

	**A4-TBA + 5% thrombin**		**ab4-TBA + 5% thrombin**		**TBA + 5% thrombin**		**TBA-ab4 + 5% thrombin**		**TBA-A4 + 5% thrombin**	
	ΔG_{diss} 298K [kJ/mol]	error	ΔG_{diss} 298K [kJ/mol]	error	ΔG_{diss} 298K [kJ/mol]	error	ΔG_{diss} 298K [kJ/mol]	error	ΔG_{diss} 298K [kJ/mol]	error
**G1**			22.77	4.88	24.65	1.92	24.69	57.53	26.99	49.32
**G6**	22.92	40.56			25.23	7.53			27.96	68.52
**G10**			23.50	25.99	24.81	24.38	27.44	62.09	26.99	49.32
**G15**	23.88	38.31	22.93	21.00	25.82	24.99			28.76	64.89
**G2**	23.62	42.62	22.24	42.17						
**G5**	21.87	35.78							27.45	62.74
**G11**	23.62	42.62			25.76	52.01				
**G14**	21.87	35.78	23.06	32.15	24.08	0.87			27.45	62.74

G2 and G5 of A4-TBA overlap over the whole temperature range, G5 and G14 of A4-TBA and TBA-A4 overlap over the whole temperature range used for fitting
